# A combined experimental and first-principle study on the oxidation mechanism of super austenitic stainless steel S32654 at 900 °C

**DOI:** 10.1038/s41598-017-00903-4

**Published:** 2017-04-13

**Authors:** Nan Dong, Caili Zhang, Huabing Li, Binbin Zhang, Peide Han

**Affiliations:** 1 Key Laboratory of Interface Science and Engineering in Advanced Materials, Taiyuan University of Technology, Ministry of Education, Taiyuan, 030024 China; 2 College of Materials Science and Engineering, Taiyuan University of Technology, No. 79, Yingze Street, Wanbolin District, Taiyuan, 030024 China; 3 School of Materials and Metallurgy, Northeastern University, No. 3-11, Wenhua Road, Heping District, Shenyang, 110819 China

## Abstract

A combined experimental and first-principle study on the oxidation mechanism of super austenitic stainless steel S32654 at 900 °C for a short time period (1, 3, and 5 h) in air is presented. The samples exhibit excellent oxidation resistance because of the initial and gradual formation of the denser Fe- and Cr-rich layer with increasing oxidation time. Meanwhile, the Mo-rich layer gradually forms because of the Mo diffusion, which results in the formation of the oxide layer with two distinct regions: an inner Fe- and Cr-rich layer and an outer Mo-rich layer. Density functional theory is applied to investigate the diffusion behaviour of Mo atom in the Fe-Cr-Ni/Cr_2_O_3_ interface and the effects of alloying elements (Fe, Ni, and Mn) on the Mo diffusion. The Mo originating from the alloy matrix tends to diffuse into the Cr_2_O_3_ part, thereby resulting in the formation of the continuous Mo-rich layer, which is consistent with the experimental behaviour. Moreover, the introduction of Ni to the Cr_2_O_3_ part can promote the Mo diffusion and the formation of the Mo-rich oxide layer, whereas Fe and Mn can hinder the Mo diffusion. The calculated results provide a microcosmic explanation of the experimental results.

## Introduction

Super austenitic stainless steels S32654 are primarily used in applications where increased pitting and crevice corrosion resistances are required, such as marine and offshore applications, seawater handling systems, nuclear power plant condenser tubes, waste incineration systems, and chemical-processing equipment^1–3^. Their extensive use is because of their superior corrosion resistance, enhanced mechanical property, formability, and weldability^4, 5^. However, the high Mo and Cr concentrates in S32654 steels may promote the segregation of the elements and formation of precipitated phases such as σ, χ, and Laves phase^3, 6–8^. Recently, many reported investigations on S32654 mainly focused on the hot deformation behaviour, precipitation behaviour, and corrosion resistance^9–11^.

In recent years, many studies have examined the high-temperature oxidation mechanism of the lower-Mo-content stainless steels^12–16^. Yun *et al*.^13^ examined the effect of Mo on the oxidation resistance of Fe–22Cr–0.5Mn ferritic stainless steel at 800 °C and confirmed that the lower Mo addition (<4 wt.%) effectively enhanced the oxidation stability at the initial oxidation stage up to 300 h. Buscail *et al*.^14^ demonstrated that Mo could restrain the outward diffusion of Fe and decrease the growth rate of the oxide layer, which enhanced the oxidation stability of the AISI 316 L stainless steel at 900 °C. Mo contents below 3 wt.% in the ferritic stainless steel could reduce the absorption of O_2_, prevent the outward diffusion of the metal cations and inward diffusion of anions, and enhance the oxidation resistance^16^. Furthermore, alloys with high Mo contents sustain the heavy oxidation at high temperatures because of the formation of volatile MoO_3_ species^17–20^. Perez *et al*.^17^ investigated the effect of Mo on the oxidation behaviour of AISI 304 and discovered that Mo could accelerate the oxidation rate because the evaporation of volatile MoO_3_ damaged the Cr_2_O_3_ layer. However, for high-Mo-content austenitic stainless steels such as S32654 steels, the effect of Mo on the high-temperature oxidation behaviour is inadequate. Therefore, the investigation on the high-temperature oxidation behaviour of S32654 steels is useful.

Moreover, our previous work^21^ mainly investigated the oxidation behaviour of S32654 steels at 900 °C, 1000 °C, and 1200 °C in air. At 900 °C, the samples exhibit excellent oxidation resistance because the MnMoO_4_ layer and denser Cr-rich oxide layer act as barriers to prevent the O_2_ inward diffusion and metal cations outward diffusion. At 1000 °C and 1200 °C, N_2_ in the air plays a significant role in accelerating the oxidation process. The specimens suffer from catastrophic oxidation, which is attributed to the synergistic action between the formation of discontinuous Cr_2_N and MoO_3_ volatilization. Cr_2_N restrains the formation of the denser Cr-rich oxide layer and provides a transportation channel for Mo, Mn, O, and N, thereby promoting the formation of MoO_3_ volatilization. However, the oxidation mechanism of S32654 steel at 900 °C in air during short time periods remains unclear, and the knowledge on the dynamic process of the initial formation of the oxide layer at the atomic scales is lacking. This work investigates the formation process of the oxide layer of the S32654 steel at 900 °C for a short time period (1–5 h). The microstructures and element distributions around the matrix/oxide layer interface were analysed. Then, the diffusion of Mo atom and the most stable site of Mo atom in the Fe-Cr-Ni/Cr_2_O_3_ interface structure were examined using the first-principle method by analysing the atomic structures and electronic properties. Finally, we systematically examined the effects of alloying additives (Fe, Ni, and Mn) in the Cr_2_O_3_ part on the diffusion of Mo atom.

## Results and Discussion

### Experimental results

**Figure 1:**
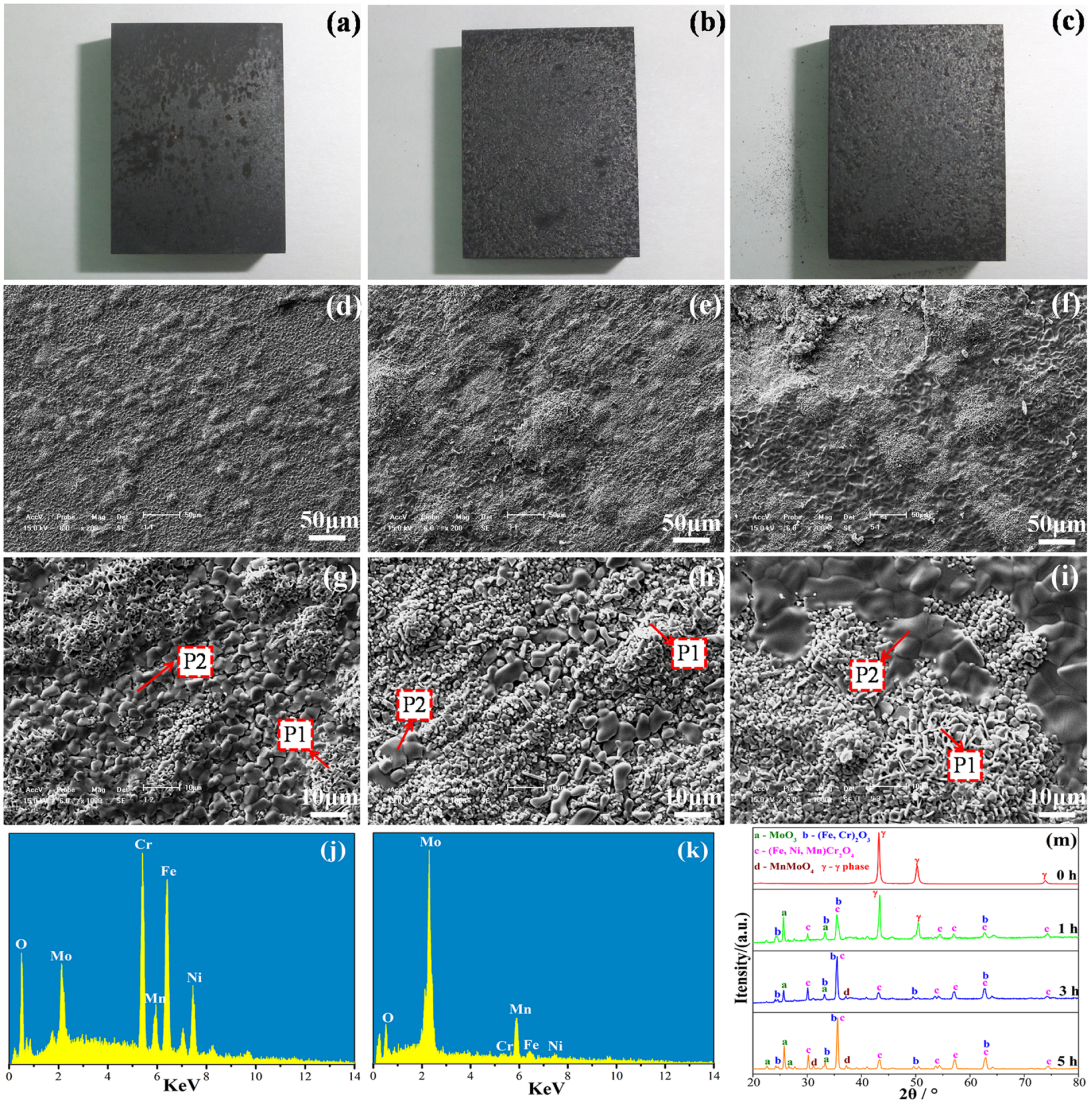
The macro morphologies of the samples after oxidation at 900 °C for 1, 3, and 5 h (**a–c**); SEM images showing the oxidized surface morphologies: 1 h (**d**,**g**), 3 h (**e**,**h**), 5 h (**f**,**i**); EDS results of the surface oxide layer: (**j**) P1, (**k**) P2; XRD results of the surface of the oxidized samples after oxidation at 900 °C (**m**).

The macro morphologies of the samples after oxidation at 900 °C for 1, 3, and 5 h show that the samples suffer from slight oxidation (Fig. 1a–c). Figure 1 also shows the low- and high-resolution SEM images of the oxidized surface morphologies of the samples after oxidation at 900 °C for 1 h (d and g), 3 h (e and h), and 5 h (f and i). The oxide layers contain two different microstructures (Fig. 1d–i): mostly smaller granular oxides (P1) and a small amount of larger grained oxides (P2). With the increase of oxidation time, the smaller granular oxides gradually increase and appear as a spinel structure at high magnification (Fig. 1g,h), and the larger grained oxides become more compact. When the oxidation time is prolonged to 5 h, the larger grained oxides (P2) become more distinct at high magnification (Fig. 1i), resulting in the formation of a compact flake attached to the surface. The EDS results show that the smaller granular oxides (P1) mainly contain Fe, Cr, Mo, Ni, and Mn, and the larger grained oxides (P2) are rich in Mo and Mn, as shown in Fig. 1j,k.

The corresponding XRD results of the surface of the oxidized samples after oxidation at 900 °C for different time periods are shown in Fig. 1m. For the non-oxidation sample, all XRD diffraction peaks correspond to the matrix (γ phases). For the oxide layer formed on the S32654 steels at 900 °C for 1 h, diffraction peaks that correspond to the γ phases are mainly observed. The new diffraction peaks indexed to the (Fe, Cr)_2_O_3_, spinel phase (Fe, Ni, Mn)Cr_2_O_4_ (as P1), and MoO_3_ (as P2) are also observed. The surface oxide layers are mainly composed of (Fe, Cr)_2_O_3_ and spinel phase (Fe, Ni, Mn)Cr_2_O_4_ with a small amount of MoO_3_. When the oxidation time increases to 3 h, the XRD peaks of the (Fe, Cr)_2_O_3_ and (Fe, Ni, Mn)Cr_2_O_4_ are notably increased, whereas the γ phase characteristic peaks are disappeared, which indicates that the amount of smaller granular oxides (P1) are gradually increased; these results are consistent with the SEM analysis. Meanwhile, the new diffraction peaks at 37.3° because of MnMoO_4_ (P2) is also observed. At 900 °C for 5 h, the (Fe, Cr)_2_O_3_, (Fe, Ni, Mn)Cr_2_O_4_, and MnMoO_4_ diffraction peaks of the oxide layer further increase and the other new diffraction peaks at 31.2° because of MnMoO_4_ is also observed. During oxidation, the initial formation of the oxide layer is (Fe, Cr)_2_O_3_ and (Fe, Ni, Mn)Cr_2_O_4_, and the MnMoO_4_ layer is gradually formed.

**Figure 2:**
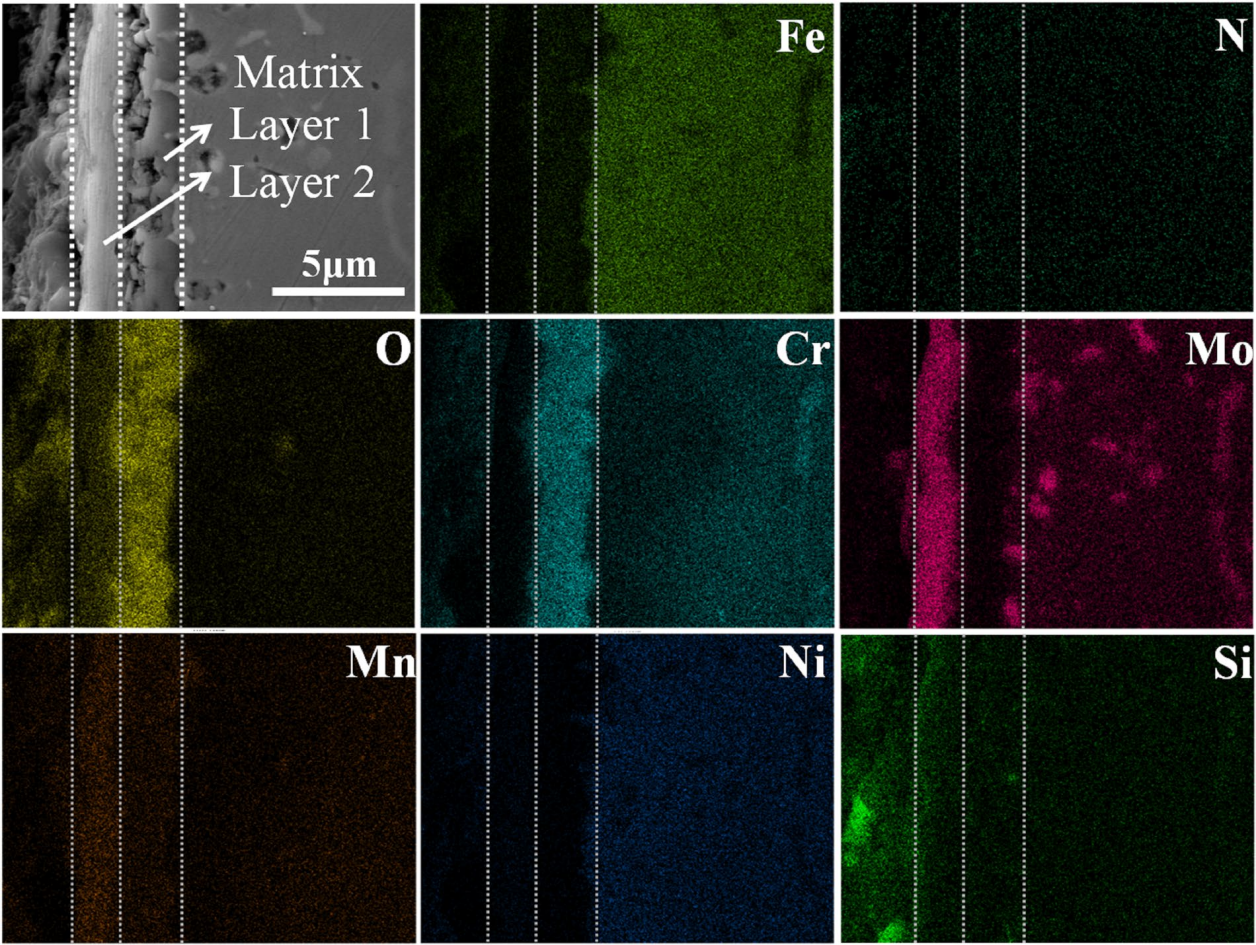
EPMA element mapping of the Fe, N, O, Cr, Mo, Mn, Ni, and Si of the cross-sections of the specimens after oxidation at 900 °C for 5 h.

Figure 2 shows the hierarchical structure of the cross-section and EPMA element mapping of the samples after oxidation at 900 °C for 5 h. A clear boundary between the matrix and the oxide layer and two distinct regions of the oxide layer are observed in the cross-sectional SEM image: an inner layer (Layer 1) and an outer oxide layer (Layer 2). The element mapping reveals that Cr, Mn, Fe, and O are enriched in Layer 1, but Mo and Mn are enriched in Layer 2. Mo is also clearly distributed in the matrix and the matrix/oxide layer interface in the form of Mo-rich precipitated phases, and it is less distributed in Layer 1. Thus, Layer 2 has a large Mo concentration possibly due to the diffusion of Mo from the matrix to the interface, gradually to the oxide layer, and finally to the surface during the long oxidation time^21^. Moreover, the matrix immediately beneath the oxide layer is denuded in Fe. These results agree with the EDS and XRD analysis.

**Figure 3:**
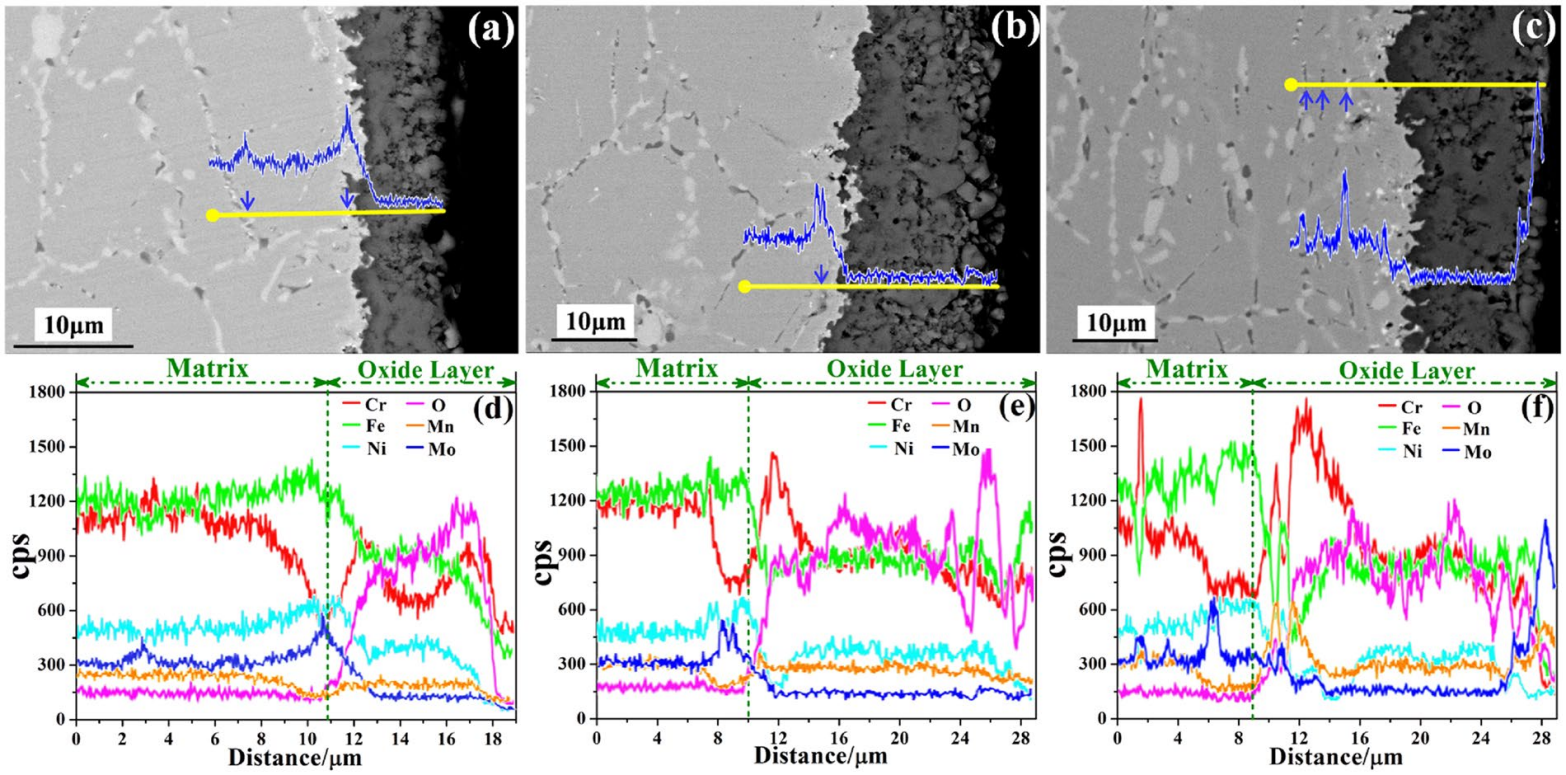
The cross-sections morphology of the specimens after oxidation at 900 °C for 1, 3, and 5 h (**a**–**c**) and the SEM–EDS line profile (**d–f**).

SEM was used to investigate the change in elemental distributions around the matrix/oxide layer interface with different oxidation times and more precisely examine the formation of the Mo-rich oxide layer during the oxidation process. Figure 3 shows the SEM images of the cross-sections morphologies of the samples after oxidation at 900 °C for 1, 3, and 5 h with the SEM–EDS line scan analyses, which were performed at the locations marked in Fig. 3(a–c). A distinct boundary between the matrix and the oxide layer is observed in the cross-sectional SEM images (a)–(c). The SEM–EDS line scan analyses reveal that according to the oxygen distributions, the thickness of the oxide layer increases from 8 μm to 19 μm and 20 μm with increasing oxidation time from 1 h to 3 h and 5 h. To more precisely examine the oxidation rate, the oxidation kinetics at 900 °C for 1, 3, and 5 h are also discussed. The weight gains per hour are 0.60 mg cm^−2^ h^−1^, 1.16 mg cm^−2^ h^−1^, and 0.92 mg cm^−2^ h^−1^ after 1, 3, and 5 h of oxidation, respectively. These results indicate that when the oxidation time increases from 3 h to 5 h, the oxide layer thickness slightly increases, and the oxidation rate is relatively low. Moreover, the Cr distribution in the oxide layer shows a distinct increase in thickness of the denser Cr-rich oxide layer with increasing oxidation time from 1 h to 3 h and 5 h. This protective layer can effectively prevent the matrix from further oxidation. These results are consistent with the aforementioned results. The Mo distribution lines and SEM images show that after 1 h of oxidation, Mo is mainly distributed at the depths of 3 μm and 11 μm, which correspond to the grain boundary areas and Mo-rich precipitated phases. Similarly, after 3 h of oxidation, Mo is mainly distributed at the depth of 8–9 μm, which corresponds to the Mo-rich precipitated phases. Therefore, after 1 and 3 h of oxidation, the oxide layers are mainly Fe- and Cr-rich oxides, and Mo is mainly distributed in the grain boundary areas and Mo-rich precipitated phases. When the oxidation time is prolonged to 5 h, Mo shows the weak peaks at the depths of 1.5 μm, 3.5 μm, and 6.5 μm, which correspond to the Mo-rich precipitated phases. Furthermore, Mo exhibits the maximum distribution near the surface of the oxide layer. Combined with the Mo distribution results in Fig. 2, the maximum Mo distribution peak near the surface can be attributed to the diffusion of Mo atoms from the matrix to the interface, gradually to the oxide layer through the grain boundary and precipitated phases, and from the oxide layer to the surface to form the continuous Mo-rich layer. Thus, a dense (Fe, Cr)_2_O_3_ oxide layer with the Mo-rich oxide layer acts as a barrier to protect the matrix from further oxidation within short time periods, but the protective effect gradually diminishes for an extended period >5 h because of the shedding as noted in previous works^21, 22^.

In conclusion, during the oxidation process, the initial formation of the oxide layer is mainly the Cr-rich oxide layer; with increasing oxidation time, the Cr-rich oxide layer becomes considerably denser, and the Mo-rich layer is gradually formed. Meanwhile, the formation mechanism of the oxide layer (Cr-rich and Mo-rich) at the atomic scales is highly limited. Thus, to investigate the formation of the Mo-rich oxide layer and more precisely explain the diffusion mechanism of Mo, the diffusion behaviour of Mo at the atomic scales is discussed in the next part using the first-principle method. The effects of Fe, Ni, and Mn, which are rich in the oxide layers, on the Mo diffusion are systematically examined.

## Calculation Results

### Diffusion behaviour of Mo atom

**Figure 4:**
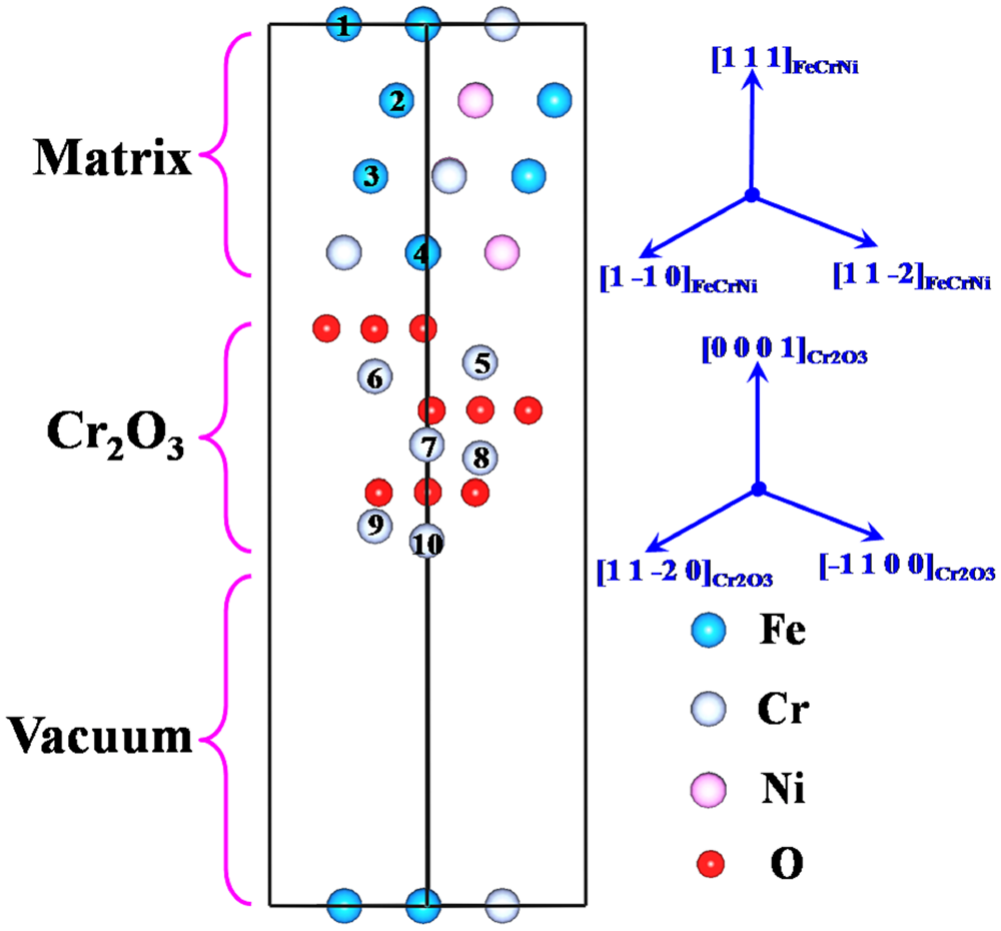
The model of Fe-Cr-Ni/Cr_2_O_3_ interface with crystalline orientation of [1 − 1 0]//[1 1 − 2 0].

The interface structure of Fe-Cr-Ni/Cr_2_O_3_ is similar to that of our previous work^23^. According to the chemical composition of S32654 steels, we introduced 4Cr and 3Ni atoms to the matrix to form the Fe-Cr-Ni/Cr_2_O_3_ interface with 22.4% Cr and 19.1% Ni (wt.%) in the Fe-base bulk. Then, we introduced Mo at different sites, as shown in Fig. 4, where numbers 1–10 denote the positions substituted by Mo atom. The realistic interface models can be used as the basis to investigate the most energetically stable or the most unstable Mo sites from an energy point and not to explore the diffusion process in ample detail.

**Figure 5:**
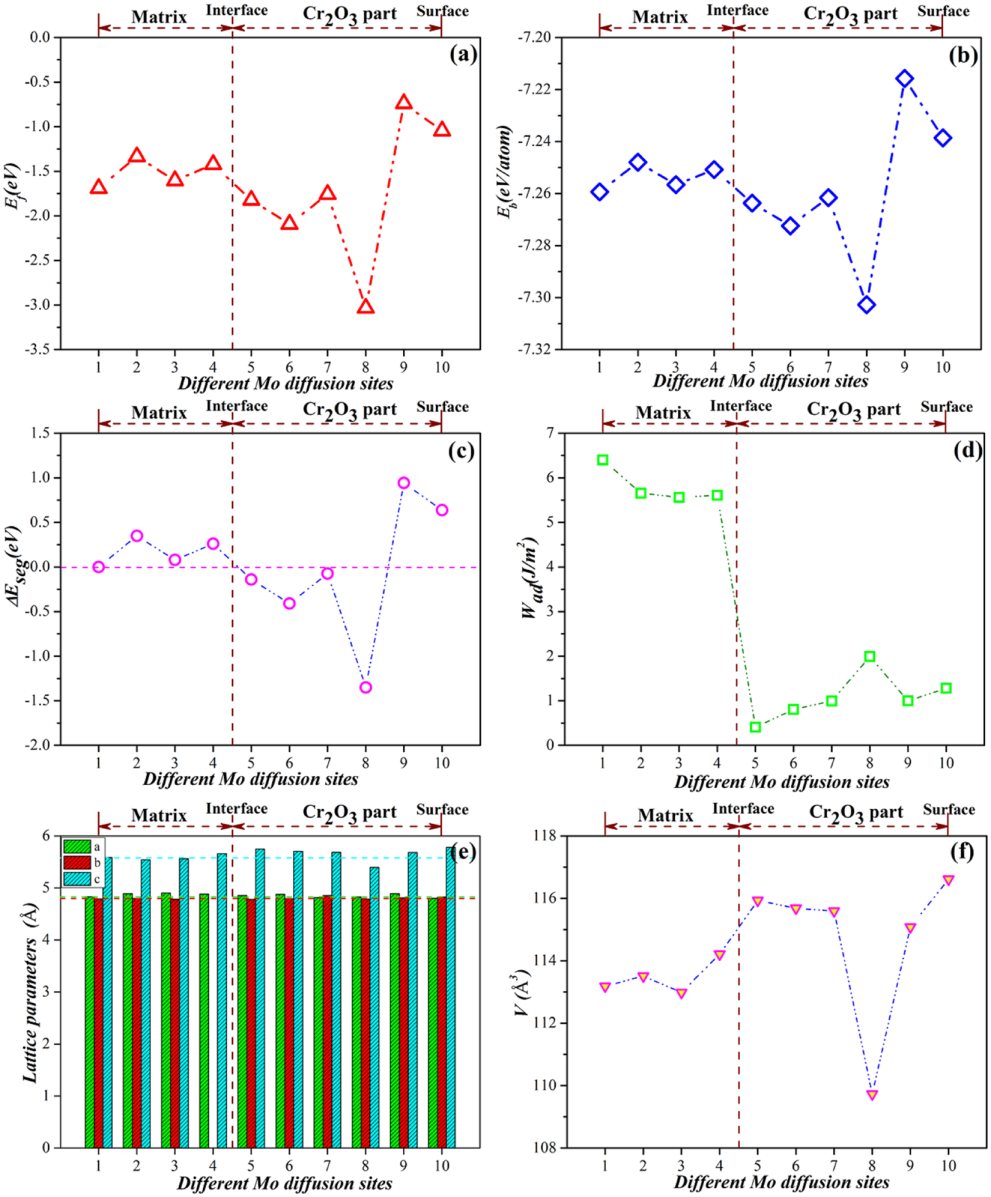
*E*_*f*_, *E*_*b*_, Δ*E*_*seg*_, *W*_*ad*_ and structure parameters of Cr_2_O_3_ part of different models in diffusion process of Mo in Fe-Cr-Ni/Cr_2_O_3_ interface, (**a**) *E*_*f*_, (**b**) *E*_*b*_, (**c**) Δ*E*_*seg*_, (**d**) *W*_*ad*_; (**e**) Lattice parameters; (**f**) Volumes.

To understand the forming abilities of different models with Mo atom at different sites, we calculate the formation energies^24^ defined in equation (1):

1 $${E}_{f}={E}_{total}-{\rm{\Sigma }}({N}_{i}{E}_{atom}^{i})$$

where *E*_*total*_ is the calculated total energy of the system, *N*_*i*_ is the number of atomic species i (i = Fe, Cr, Ni, Mo, or O), and ${E}_{atom}^{i}$ is the energy of each atom i (i = Fe, Cr, Ni, Mo, or O) in its most stable state. The most stable structures of Fe (fcc), Cr (bcc), Ni (fcc), Mo (bcc), and O_2_ are used to calculate the forming energy. The calculated formation energies are presented in Fig. 5a. All formation energies are negative, which shows that Mo atom at these sites are all easily formed. As Mo atoms are in the alloy matrix (Sites 1–4), the *E*_*f*_ values are larger than those sites of Mo atoms in the Cr_2_O_3_ part (Sites 5–8), which shows that Mo atoms are readily crystallized at Sites 5–8.

To further understand the thermodynamic stabilities of different models with Mo atom at different sites, we calculate their binding energies using equation (2):

2 $${E}_{b}=\frac{1}{\Sigma {N}_{i}}[{E}_{total}-\Sigma ({N}_{i}{E}_{iso}^{i})]$$

where ${E}_{iso}^{i}$ is the energy of isolated atom i (i = Fe, Cr, Ni, Mo, or O). ${E}_{iso}^{i}$ is calculated by putting an i (i = Fe, Cr, Ni, Mo, or O) atom in the middle of the cubic unit cell with a lattice constant of 10 Å. The calculated binding energies are shown in Fig. 5b. All binding energies are negative, which indicates that these structures are thermodynamically stable. As Mo atoms are in the alloy matrix (Sites 1–4), the values of *E*_*b*_ are all larger than those of Mo atoms in the Cr_2_O_3_ part (Sites 5–8), which shows that Sites 5–8 are more stable, and the interactions among the atoms in these structures are much stronger than those in Sites 1–4.

Then, we calculate the heats of segregation (Δ*E*_*seg*_)^25, 26^ of different models with Mo atom at different sites. Δ*E*_*seg*_ is defined in equation (3):

3 $${\rm{\Delta }}{E}_{seg}={E}_{f}^{M}-{E}_{f}^{bulk}$$

where M is the interface structure with Mo atom at the special diffusion site (Sites 1–10); ${E}_{f}^{M}$ and ${E}_{f}^{bulk}$ are the formation energies of Mo in the M site and Fe bulk (Site 1 in our case), respectively. A negative Δ*E*_*seg*_ indicates that Mo tends to segregate to specific sites. Here, Δ*E*_*seg*_ was calculated to determine the most energetically stable or most unstable Mo sites and not to explore the diffusion process in ample detail.

To examine the interfacial adhesive properties of different models, the works of adhesion (*W*_*ad*_)^27^ are further calculated based on equation (4):

4 $${W}_{ad}=({E}_{t}^{{Fe}-{Cr}-{Ni}}+{E}_{t}^{C{r}_{2}{O}_{3}}-{E}_{t}^{{Fe}-{Cr}-{Ni}/C{r}_{2}{O}_{3}})/2A$$

where ${E}_{t}^{{Fe}-{Cr}-{Ni}/C{r}_{2}{O}_{3}}$ is the total energy of the Fe-Cr-Ni/Cr_2_O_3_ interface structure; ${E}_{t}^{{Fe}-{Cr}-{Ni}}$ and ${E}_{t}^{C{r}_{2}{O}_{3}}$ are the energies of the isolated Fe-Cr-Ni matrix and Cr_2_O_3_ slabs, respectively; and A is the interfacial area of the unit cell.

The calculated values of Δ*E*_*seg*_ are shown in Fig. 5c. When the Mo atoms are in the alloy matrix (Sites 1–4), all values of Δ*E*_*seg*_ are positive, which implies that Mo atoms do not tend to segregate to these sites. When the Mo atoms are in the Cr_2_O_3_ part (Sites 5–8), Δ*E*_*seg*_ are negative, which shows that Mo atoms tend to segregate to the oxide layer and replacing a Cr atom near the subsurface (Site 8) is predicted to be the most stable Mo diffusion site with Δ*E*_*seg*_ of −1.35 eV. When the Mo atoms are at the surface of the oxide layer (Site 9–10), the values of Δ*E*_*seg*_ increase remarkably, which indicates that the oxide layer surface is the most unstable diffusion site of Mo atoms. Thus, in terms of energies, Mo atom tends to diffuse from the site in the alloy bulk (Sites 1–4) to the subsurface of the Cr_2_O_3_ layer, and the surface of the oxide layer is the most unstable diffusion site. Figure 5d displays *W*_*ad*_ and shows that when Mo atoms diffuse from the alloy bulk (Sites 1–4) to the Cr_2_O_3_ part (Sites 5–10), the values of *W*_*ad*_ significantly decrease. Hence the mechanical properties of the interfaces are weakened when Mo atoms are in the oxide layer. Thus, the adhesion of the matrix/oxide layer interface appreciably weakens, and the protective effect should diminish because of the detachment of the oxide layer. Our calculations further confirm our experimental results in Section 3.1.

The lattice parameters and volumes of the Cr_2_O_3_ part of different models in the diffusion process of Mo are calculated and are listed in Fig. 5e,f. The lattice parameters show that when the Mo atoms are in the alloy matrix (Sites 1–4), the values of lattice parameters a, b, and c remain unchanged, and the volumes slightly vary. When the Mo atoms are in the Cr_2_O_3_ part (Sites 5–10), the values of a and b are also nearly unchanged. However, c distinctly increases, and the volumes are relatively larger than those of in Sites 1–4. When the Mo atoms are in the oxide layer, the oxide layer along the Z-axis expands. When the Mo atom is in Site 8, the value of c and volume are minimized, which shows that the interactions among the atoms are maximized (corresponding to the most stable structure). In brief, when Mo atoms diffuse from the matrix to the oxide part, the lattice distortion causes the volume expansion and density decrease of the oxide part, which promotes the diffusion of Mo atoms.

Figure 5e,f show that when Mo atoms are in the matrix, a limited effect on the oxide layer is detected. To understand the bonding strength among different atoms in the oxide layer when the Mo atoms are in the oxide part (Sites 5–10), the atomic transfer charges of the Cr, O, and Mo atoms of different models were calculated using Mulliken population analysis^28^ as shown in Table 1. The values of the Mo atoms (0.93, 1.03, 0.89, 0.78, 0.76, and 0.55) increase compared with those of the corresponding Cr atoms (0.73, 0.76, 0.65, 0.67, 0.53, and 0.50). Concurrently, the electronegativities of the O atoms next to the Mo atoms are higher and supported by the transfer charges (−0.54, −0.57, −0.55, −0.57, −0.56, and −0.53), which indicates that the interactions between Mo atoms and O atoms strengthen when Mo atoms are in the oxide part. The values of the Cr atoms next to Mo atoms decrease, which shows that the interactions between Cr and O atoms are weaker. These results indicate that when Mo atoms diffuse into the oxide part, they generate a strong positive electric field that offers a strong attractive power for O atoms, whereas the interactions between Cr and O atoms near Mo atoms weaken and cause a decrease in density of the oxide part, which promotes the diffusion of Mo atoms.

**Table 1:** Atomic transfer charges of the Cr, O, and Mo atoms in the Cr_2_O_3_ part when Mo atoms are in the oxide part (Sites 5–10).

Site 5		Site 6		Site 7		Site 8		Site 9		Site 10	
Atom	Transfer charge (e)	Atom	Transfer charge (e)	Atom	Transfer charge (e)	Atom	Transfer charge (e)	Atom	Transfer charge (e)	Atom	Transfer charge (e)
O	−0.48	O	−0.49	O	−0.46	O	−0.45	O	−0.46	O	−0.46
O	−0.53	O	−0.53	O	−0.51	O	−0.51	O	−0.51	O	−0.51
O	−0.49	O	−0.54	O	−0.51	O	−0.44	O	−0.51	O	−0.51
**Mo**	**0.93**	**Cr**	**0.65**	Cr	0.63	Cr	0.65	Cr	0.74	Cr	0.73
**Cr**	**0.71**	**Mo**	**1.03**	Cr	0.76	Cr	0.69	Cr	0.76	Cr	0.77
**O**	−**0.54**	**O**	−**0.57**	O	−0.53	O	−0.53	O	−0.54	O	−0.51
O	−0.54	O	−0.48	O	−0.55	O	−0.54	O	−0.50	O	−0.51
O	−0.53	O	−0.48	**O**	−**0.55**	O	−0.52	O	−0.50	O	−0.51
Cr	0.65	Cr	0.65	**Mo**	**0.89**	**Cr**	**0.58**	Cr	0.66	Cr	0.68
Cr	0.67	Cr	0.68	**Cr**	**0.63**	**Mo**	**0.78**	Cr	0.64	Cr	0.68
O	−0.51	O	−0.52	O	−0.56	**O**	−**0.57**	O	−0.53	O	−0.52
O	−0.54	O	−0.53	O	−0.54	O	−0.56	O	−0.52	O	−0.51
O	−0.51	O	−0.52	O	−0.53	O	−0.53	**O**	−**0.56**	**O**	−**0.53**
Cr	0.53	Cr	0.56	Cr	0.53	Cr	0.53	**Mo**	**0.76**	**Cr**	**0.46**
Cr	0.50	Cr	0.49	Cr	0.49	Cr	0.74	**Cr**	**0.38**	**Mo**	**0.55**

### 3.2.2 Effect of Fe, Ni and Mn on the diffusion of Mo atom

In the past, EDS, XRD, and EPMA analyses demonstrate that the oxide layers are rich in Fe, Cr, Ni, and Mn. Thus, these alloying elements can affect the diffusion of Mo atoms in the Fe-Cr-Ni/Cr_2_O_3_ interface. Here, we examine the potential effects of Fe, Ni, and Mn on the diffusion of Mo in the Cr_2_O_3_ part (from Site 5 to Site 10); each additive replaced the Cr atom in the Cr_2_O_3_ part.

**Figure 6:**
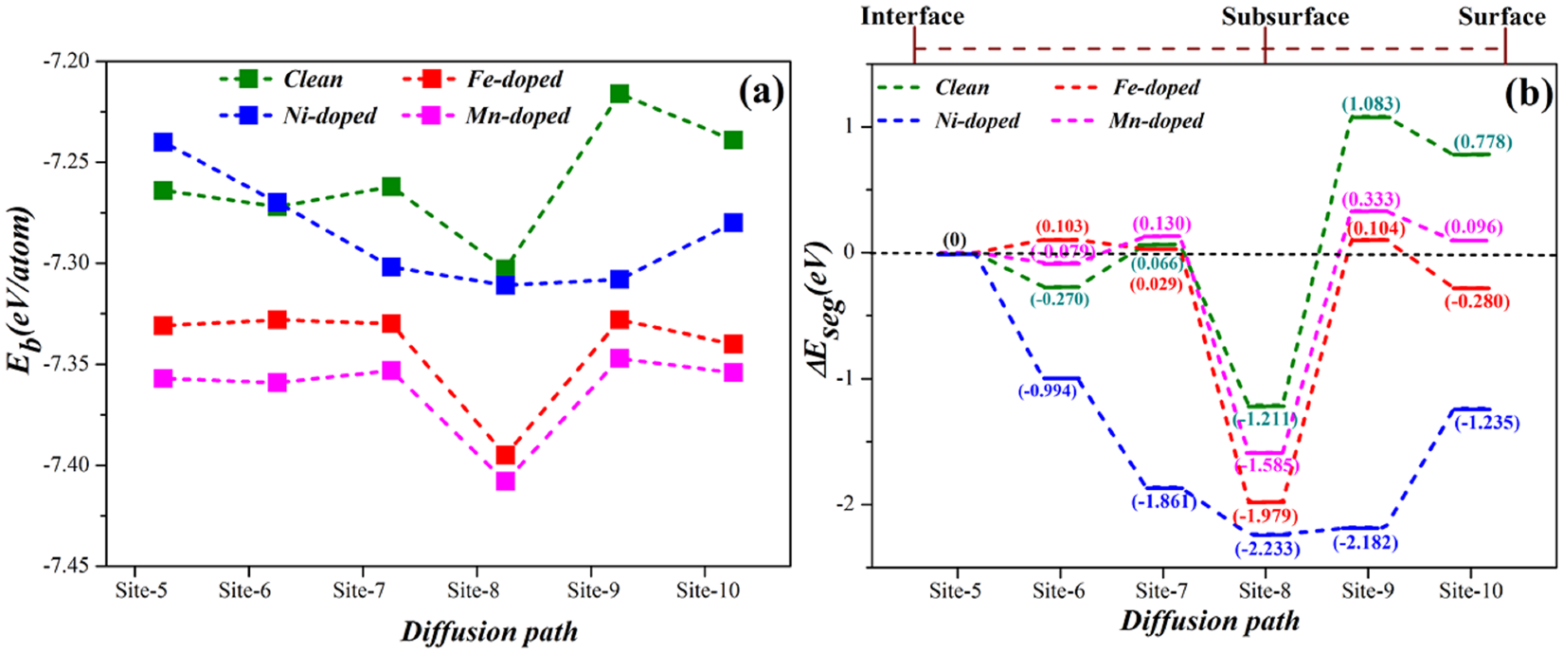
*E*_*b*_ and Δ*E*_*seg*_ of different models in diffusion process of Mo in Cr_2_O_3_ part of Fe-Cr-Ni/Cr_2_O_3_ interface with Fe, Ni, and Mn doped in Cr_2_O_3_ part, (**a**) *E*_*f*_, (**b**) Δ*E*_*seg*_.

To understand the thermodynamic stabilities of different models of Mo atom at different sites (Sites 5–10) of Fe-, Ni-, and Mn-doped Fe-Cr-Ni/Cr_2_O_3_ structures, we calculate their binding energies as shown in Fig. 6a. All binding energies are negative, which indicates that these structures are stable. Among different systems, site 8 is constantly most stable and shows that the Mo atom tends to settle at the subsurface of the structure. For different alloying-element-doped systems, when the Mo atom is retained at the same site, *E*_*b*_ approximately holds the following order: Clean > Ni-doped > Fe-doped > Mn-doped, which indicates that the Mn-doped system is the most stable. These results are in consistent with the results in Fig. 2.

We calculate Δ*E*_*seg*_ of Mo in different sites (Sites 5–10) of Fe-, Ni-, and Mn- doped Fe-Cr-Ni/Cr_2_O_3_ structures, as shown in Fig. 6b. The introduction of Fe, Ni, and Mn to the Cr_2_O_3_ part does not change the diffusion behaviour of the Mo atom: the Mo atom also tends to segregate at Site 8; the relative difficulty of Mo diffusion varies in different degrees. When Fe and Mn atoms are introduced, the large energy barrier must be overcome to diffuse from Site 5 to Site 8. Thus, the Mo diffusion is relatively difficult compared with that in the structure of the clean interface. When the Ni atom is introduced to the oxide part, the Mo atom spontaneously diffuses from Site 5 to Site 8, and the energies released in the diffusion process is 2.233 eV, which shows that the diffusion has a larger driving force than others. Thus, in terms of energies for Fe-, Ni-, and Mn-doped interface structures, the Mo atom tends to diffuse from Site 5 to Site 8, and the surface of the oxide layer (Sites 9 and 10) is the most unstable diffusion site. Furthermore, Ni can promote the Mo diffusion and increase the growth rate of the Mo-rich oxide layer, whereas Fe and Mn can hinder the Mo diffusion.

Table 2 lists the atomic transfer charges for all Cr, O, Mo, and Fe(Ni, Mn) atoms in the Cr_2_O_3_ part of the most stable structure (Site 8) in different interfaces. For a clean interface, the Mo atom at Site 8 generates a large positive electric field that offers a strong attractive power for O atoms, whereas the interactions between Cr atoms and O atoms near the Mo atoms weaken and cause a decrease in density of the oxide part, which promotes the diffusion of Mo atoms. For the Fe- and Mn-doped systems, the transfer charges of the Mo atoms slightly change compared with that of the clean interface. The values of O atoms near the Mo atoms are almost unchanged. These results indicate the negligible effects of Fe and Mn doping on the interactions between Mo atoms and O atoms. For the Ni-doped system, the value of Mo atom is 0.66, that of O atom is 0.53, and that of Ni atom represented Cr atom decreasing to 0.51. The Mo atom generates a weaker positive electric field. This assumption is proven to be correct by the less negative values of O atoms that follow Mo. Therefore, the interactions between Mo (Ni) atoms and O atoms are weakened. These phenomena show that Ni doping will cause a more obvious decrease in density of the oxide part, which will promote the diffusion of Mo atoms.

**Table 2:** Atomic transfer charges of the Cr, O, Mo, and Fe(Ni, Mn) atoms in the Cr_2_O_3_ part of the most stable structure (Site 8) in different interfaces.

Clean		Fe–doped		Ni–doped		Mn–doped	
Atom	Transfer charge (e)	Atom	Transfer charge (e)	Atom	Transfer charge (e)	Atom	Transfer charge (e)
O	−0.51	O	−0.52	O	−0.52	O	−0.52
O	−0.45	O	−0.44	O	−0.45	O	−0.45
O	−0.44	O	−0.44	O	−0.45	O	−0.45
Cr	0.65	Cr	0.68	Cr	0.74	Cr	0.67
Cr	0.69	Cr	0.72	Cr	0.69	Cr	0.69
O	−0.53	O	−0.53	O	−0.53	O	−0.54
O	−0.54	O	−0.54	O	−0.54	O	−0.52
O	−0.52	O	−0.55	O	−0.55	O	−0.52
**Cr**	**0.58**	**Fe**	**0.59**	**Ni**	**0.51**	**Mn**	**0.73**
**Mo**	**0.78**	**Mo**	**0.77**	**Mo**	**0.66**	**Mo**	**0.77**
**O**	−**0.57**	**O**	−**0.56**	**O**	−**0.53**	**O**	−**0.55**
O	−0.56	O	−0.56	O	−0.54	O	−0.56
O	−0.53	O	−0.53	O	−0.53	O	−0.53
Cr	0.53	Cr	0.48	Cr	0.47	Cr	0.47
Cr	0.74	Cr	0.74	Cr	0.70	Cr	0.71

## Conclusions

In this work, we have systematically determined the oxidation mechanism of super austenitic stainless steel S32654 at 900 °C for a short time period (1–5 h) in air using experimental and first-principle methods. The samples exhibit excellent oxidation resistance because of the initial and gradual formation of the denser Fe- and Cr-rich layer with increasing oxidation time. This layer acts as a barrier that effectively protects the matrix from further oxidation. Meanwhile, the Mo-rich layer is gradually formed because Mo atoms diffuse from the matrix to the oxide part. After 5 h, the oxide layer has two distinct regions: an inner Fe- and Cr-rich layer and an outer Mo-rich layer. Density functional theory is used to investigate the diffusion behaviour of Mo atoms in the Fe-Cr-Ni/Cr_2_O_3_ interface, as well as the effects of alloying elements (Fe, Ni, Mn) on the Mo diffusion. Based on the calculated Δ*E*_*seg*_, during the oxidation process, Mo atoms from the alloy matrix tend to diffuse into the Cr_2_O_3_ part, which is consistent with the experimental behaviour. We elucidate the experimental observations related to the oxidation of S32654 steels and show that the introductions of Ni atom to the Cr_2_O_3_ part can promote the Mo diffusion and formation of the Mo-rich oxide layer, whereas Fe and Mn can slow the Mo diffusion.

### Experiment and calculation details

The steel in this work is a newly developed super austenitic stainless steel S32654. Its chemical composition (wt.%) is 23.36% Cr, 21.75% Ni, 7.46% Mo, 3.40% Mn, 0.015% C, 0.46% N, 0.22% Si, 0.46% Cu, 0.007% P and 0.001% S, with Fe as the balance. The samples were solution-treated at 1200 °C for 1 h and subsequently water-quenched to eliminate the precipitation of the second phase. The specimens for the oxidation test with the dimensions of 20 mm × 15 mm × 6 mm were carefully ground using SiC paper to 2000 grit and exposed to 900 °C in air for 1, 3, and 5 h. The surface morphologies of the oxide layers were observed using a scanning electron microscope (SEM) with an energy dispersive spectrometer (EDS). The constituent phases of the oxide layer were determined using X-ray diffraction (XRD), and the XRD analysis was directly performed on the surface of the oxidized samples. The morphology of the cross-sections and element distributions were determined using an electron probe micro-analyser (EPMA). Atomistic calculations were conducted using the first-principle method with the CASTEP code. The calculated parameters and details are similar to our previous work^23, 29, 30^.
